# The diagnostic pitfalls of lumbar disc herniation---- malignant sciatic nerve tumour: two case reports and literature review

**DOI:** 10.1186/s12891-021-04728-1

**Published:** 2021-10-05

**Authors:** Li Zhao, Junqiang Wei, Chenguang Wan, Shuhong Han, He Sun

**Affiliations:** 1 Department of Spine Surgery, Affiliated Hospital of Chengde Medical College, Chengde, 067000 Hebei China; 2 Department of Traumatic Orthopedics, Affiliated Hospital of Chengde Medical College, Chengde, Hebei 067000 China; 3 Department of Neurosurgery, Tianjin First Central Hospital, Tianjin, 300000 China

**Keywords:** Lumber disc herniation, Sciatic nerve, Misdiagnosis, Tumour

## Abstract

**Background:**

Sciatica pain is a typical symptom of lumbar disc herniation (LDH), but some neurogenic and malignant tumours surrounding the sciatic nerve can also cause similar symptoms. These tumours are often misdiagnosed or even mistreated as LDH in clinical practice.

**Case presentation:**

In our clinical practice, we found two patients with malignant tumours who were misdiagnosed with LDH. One patient complained of pain and numbness in the right lower limb. The primary diagnosis was LDH, and the patient underwent posterior lumbar interbody fusion surgery. After the operation, the symptoms were not alleviated. Then, diffuse large B-cell lymphoma involving the soft tissue and the sciatic nerve was identified. Another patient who manifested with radiating pain in the right lower limb was diagnosed with LDH at Chengde Central Hospital. He received regular conservative treatment for approximately 6 months, but his symptoms were not relieved, and then he was referred to our hospital. A malignant peripheral nerve sheath tumour (MPNST) of the sciatic nerve was diagnosed, and he received cisplatin (DDP) chemohyperthermia.

**Conclusions:**

Descriptions of tumour lesions involving the sciatic nerve and misdiagnosed as LDH in the literature are rare. In the reported literature, 7 patients were misdiagnosed with LDH, and all patients presented with sciatica. Among them, 4 patients only received surgical treatment, 1 patient only underwent neurolysis, and 2 patients received both surgical and chemotherapy treatment. Their low incidence and similar clinical manifestations to LDH make malignant tumours involving the sciatic nerve easy to misdiagnose. When the clinical symptoms and signs are inconsistent with the imaging findings, we need to be aware of non-discogenic sciatica, including tumours involving the sciatic nerve. Furthermore, tumours that grow near the exit of the sciatic notch may be misdiagnosed because of their deeper location and because they are covered with gluteal muscles. Sometimes sciatica caused by sciatic nerve tumours is only distal, without any radicular distribution. This pain is more severe than that caused by LDH, and this pain is not related to the position of the lumbar spine. Thus, it is beneficial to perform a detailed physical examination of the sciatic nerve to avoid this kind of misdiagnosis.

## Background

Lumbar disc herniation (LDH) often occurs because of intervertebral disc degeneration, and trauma is one of the important factors in its pathogenesis. In addition to degeneration and trauma, genetic and developmental abnormalities are also related to LDH [1, 2]. Typical clinical manifestations of LDH are low back pain, sciatica, muscle weakness, sensory deficits, and nerve root tension sign [1, 3]. However, sciatic nerve-derived tumours and surrounding neoplasms also show similar symptoms. For such patients, sciatica caused by tumours is often erroneously diagnosed as LDH, and radiological imaging might show mild to moderate disc disease. Furthermore, these patients always receive the wrong treatment. The common clinical manifestations of these patients usually include sciatica, followed by atrophy, tenderness on deep intragluteal palpation, positive Tinel’s sign, positive Laseque’s sign, plantar flexion weakness, and a decreased ankle reflex [4–14]. It is worth considering that when patients present with unrelenting pain at rest, the possibility of tumours should be borne in mind, and proper imaging techniques should be used [10, 11, 14, 15]. In 2019, Fernando Guedes et al. [5] reported a patient with low-grade sarcoma who was misdiagnosed with LDH. The authors indicated that the key point for avoiding misdiagnosis is the consistency between the clinical manifestations and radiological imaging findings, especially on magnetic resonance imaging (MRI) [5]. Due to the rarity of malignant tumours involving the sciatic nerve, they are easily misdiagnosed as LDH. In our clinical practice, we found two cases of malignant tumours involving the sciatic nerve that were misdiagnosed as LDH. Inappropriate treatment caused by misdiagnosis may lead to legal disputes, especially delayed treatment of malignant tumours, which has a serious impact on patients. Thus, we suggest that for patients with persistent sciatica and only mildly altered or normal lumbar images, other aetiologies must be suspected, including those with an extraspinal origin. Below, we illustrate these two cases and a review the reported cases in the literature.

## Case presentation

### Case 1

**Fig. 1:**
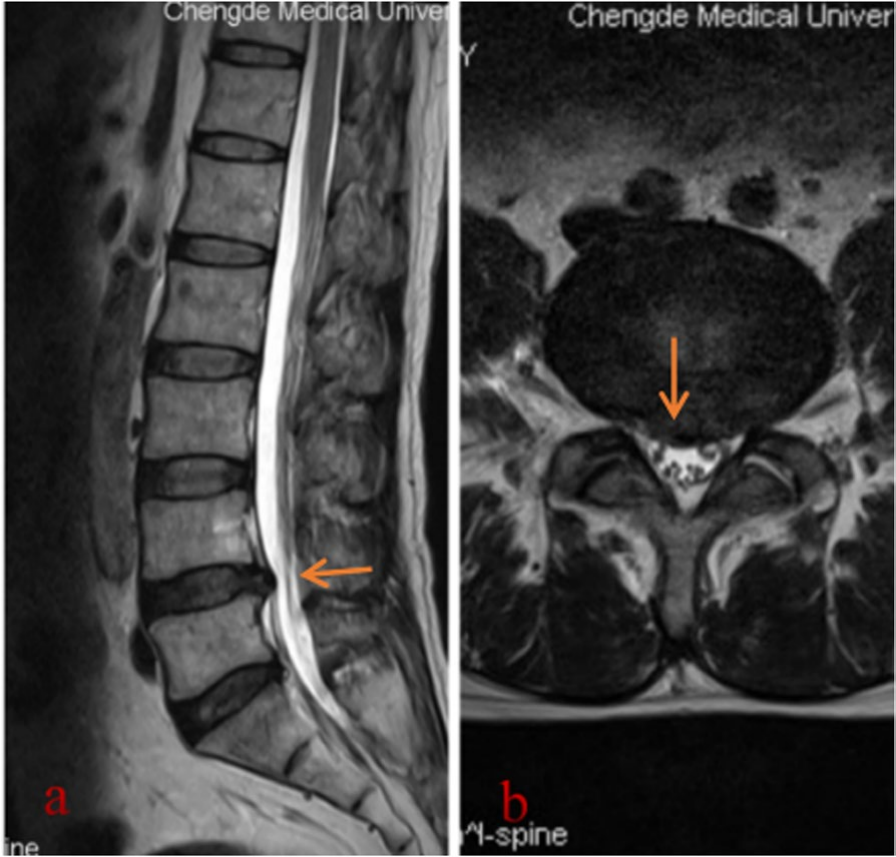
Lumbar MRI in case 1. **a** Sagittal MRI shows L4-5 intervertebral disc herniation and compression of the dural sac (arrow). **b** Axial MRI shows lumbar disc herniation and compression of the L5 nerve root (arrow)

**Fig. 2:**
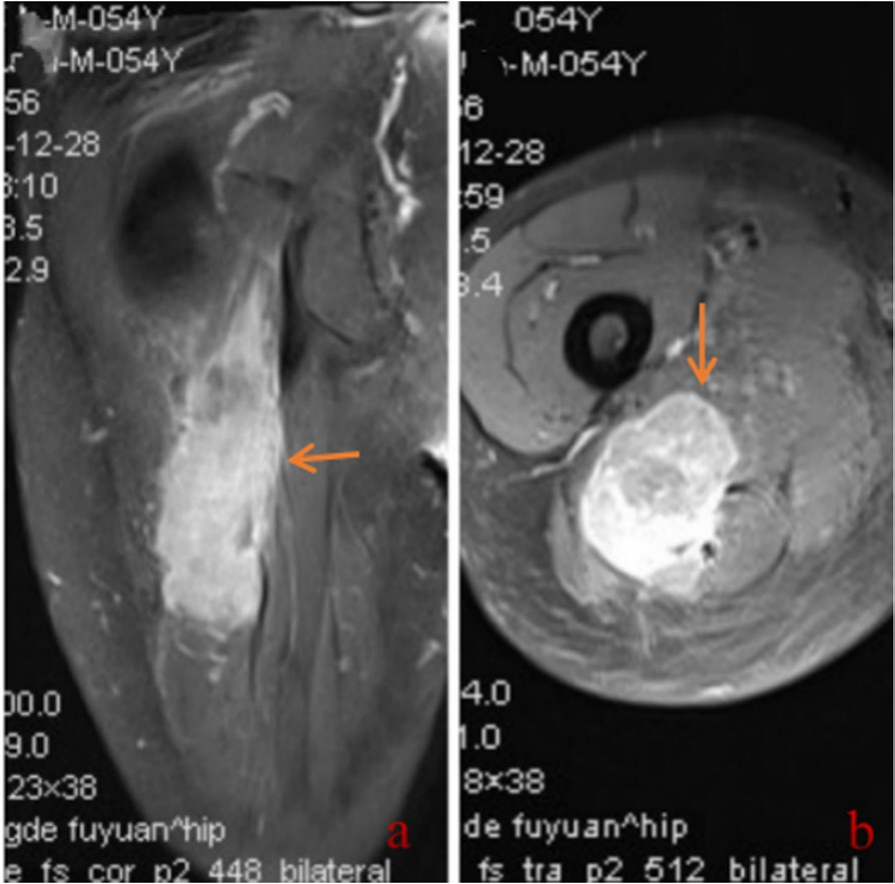
Gluteal MRI in case 1. **a** Coronal MRI shows that the right thigh tumour appeared as an intramuscular mass with inhomogeneous vascularization (arrow). **b** T2-weighted axial MRI shows a mass with high signal intensity (arrow)

**Fig. 3:**
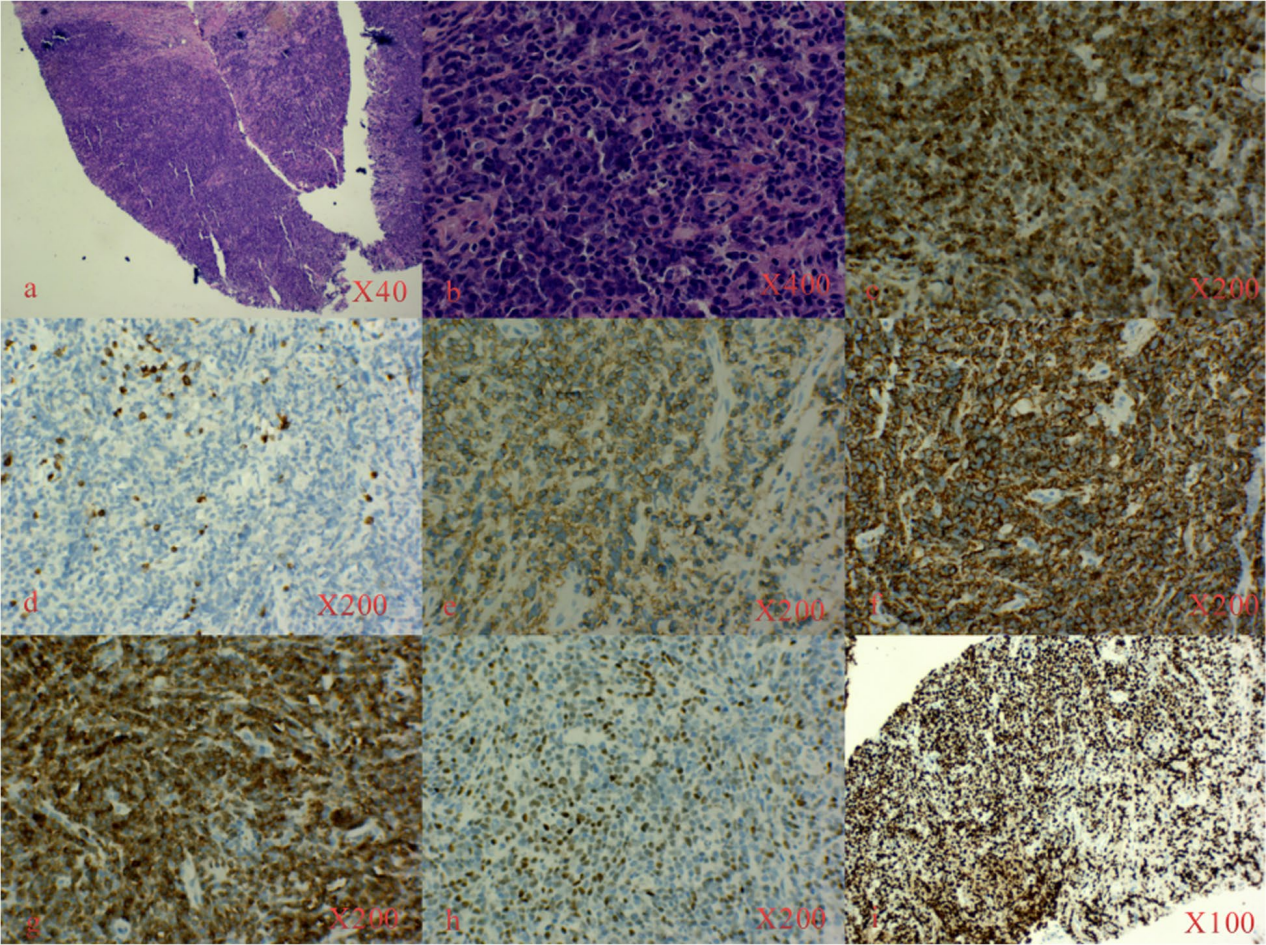
HE staining and immunohistochemistry staining in case 1. **a**, **b** HE staining shows that the lymphoma cells were large and arranged in a diffuse pattern. **c**-**i** The tumour sections were positive for Bcl-2 (**c**), CD3 (**d**), CD19 (**e**), CD20 (**f**), CD79α (**g**), c-myc (**h**) and Ki67 (**i**) by immunohistochemistry staining

A 54-year-old man complained of pain and numbness in the right lower limb for 6 months. This intermittent intense pain and numbness could be slightly relieved by supine positioning and hip overextension and was aggravated after overuse. The visual analogue scale (VAS) score of pain was 6/10 at the first visit to the clinic, and the pain did not wake the patient up from sleep during the night. Physical examination revealed a decreased tingling sensation of the right hip, right posterolateral leg and foot skin, and there was slight tenderness in the lumbar 4-5 intervertebral space. In addition, a grade 3/5 weakness of the extensor hallucis longus and a positive straight leg raise test were found. The muscular tension, knee reflex and Achilles tendon reflex were normal, and the patient had a negative Babinski sign, Chaddock sign, Gordon sign and Oppenheim sign. Lumbar MRI examinations were performed in the clinic, and lumbar 4-5 intervertebral disc herniation were found (Fig. 1). Based on the patient complaints, physical signs and radiological examination, the patient was primarily diagnosed with LDH and admitted to the spine surgery department on 8 November 2020. After admission, written consent was obtained from the patient, and posterior lumbar discectomy and interbody fusion surgery were performed. During the operation, L4-5 disc herniation was found, and the L5 nerve root was compressed. After surgery, 2 g cefazolin and 60 mg ketorolac tromethamine were given intravenously every 12 h for 3 to 4 days. However, there was no relief of the lower limb pain 2 weeks after the operation. Considering that the lower limb pain was caused by nerve root oedema, Neurotropin and imrecoxib were administered. Unfortunately, the symptoms gradually worsened. The pain VAS score was 7/10. Then, a multidisciplinary team (MDT) was organized on 25 December 2020. The patient received a detailed physical examination, and a palpable mass near the buttocks was found. The mass was approximately 50 × 240 mm and hard and led to poor activity, pain that radiated from the right lower extremity and a positive Tinel’s sign. At this time, the patient received an electromyogram (EMG) examination, enhanced MRI scan of the tumour and positron emission tomography-computed tomography (PET-CT) scan. EMG indicated that the conduction velocities of the sensory and motor nerves of the sciatic nerve were slow. An enhanced MRI scan of the right thigh tumour revealed an intramuscular mass measuring 58 × 36 × 90 mm with inhomogeneous vascularization. T1-weighted MRI showed a nonuniform low-intensity mass in the gluteal region, while T2-weighted MRI showed a mass with high signal intensity (Fig. 2). PET-CT indicated a 54 × 51 × 240 mm soft tissue tumour that was characterized by “fusiform” expansion. The maximum standardized uptake value (SUVmax) of the tumour was 38.6. At this time, the tumour was primarily diagnosed as soft tissue sarcoma. Furthermore, we think that the radiating pain in the lower extremities felt by the patient was attributable to this tumour. Then, the patient underwent tumour percutaneous biopsy on 3 January 2021. Haematoxylin-eosin (HE) staining showed that the large lymphoma cells were arranged in a diffuse pattern and that the nuclei of the cells were round (centroblast-like) or multilobulated, and no necrotic regions or nuclear fission was observed. Immunohistochemical staining revealed the following: CD20(+), CD19(+), CD3(+), CD79α (+), c-myc(+), ki67(+), multiple myeloma oncogene 1 (MUM1) (+), B-cell lymphoma-2 (Bcl-2) (+), and Bcl-6 (+) (Fig. 3). The diagnosis of diffuse large B-cell lymphoma with soft tissue involvement was established by histopathological examination. Finally, the patient was transferred to the haematology department for further treatment. After 1 cycle of rituximab, cyclophosphamide, hydroxydaunomycin, oncovin, and prednisone (R-CHOP), the tumour shrank, and the symptoms were alleviated.

### Case 2

**Fig. 4:**
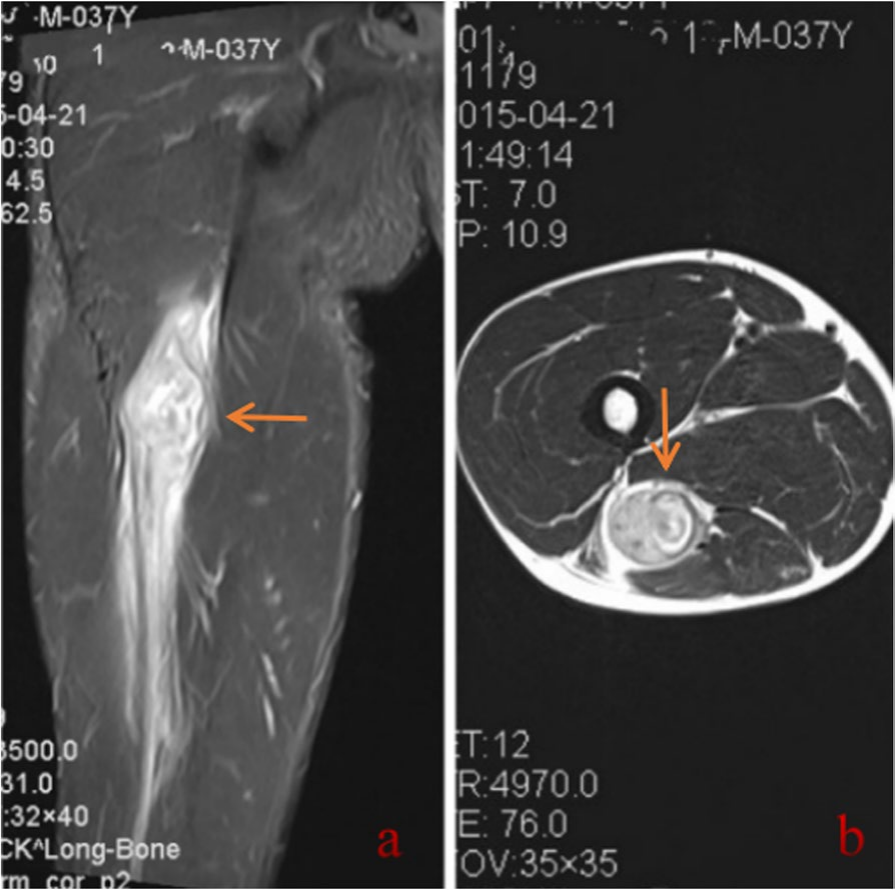
Gluteal MRI in case 2. **a** Coronal MRI shows a fusiform tumour in the femoral region (arrow). **b** T2-weighted axial MRI shows a mixed low- and high-intensity signal (arrow)

**Fig. 5:**
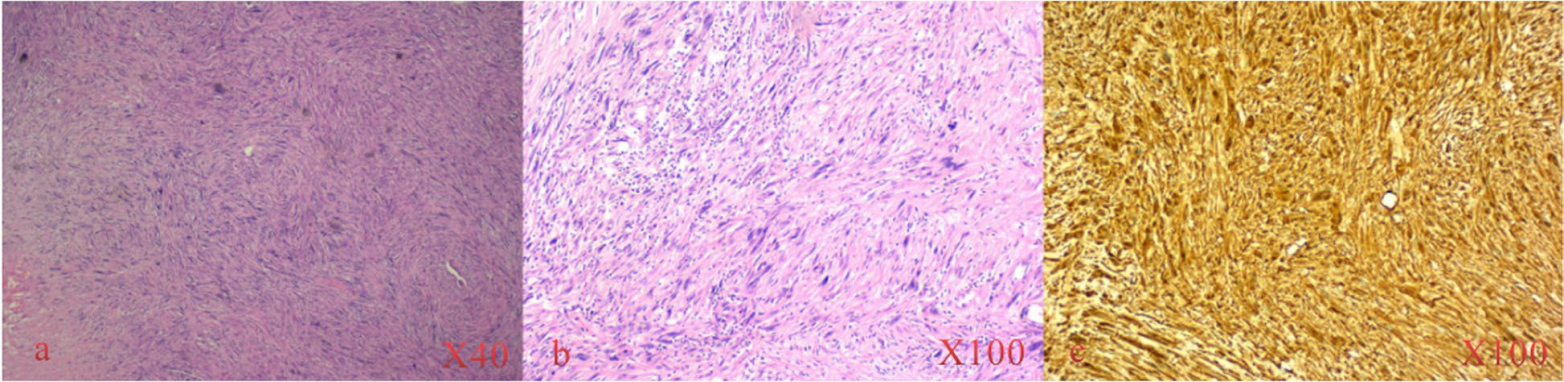
HE staining and immunohistochemistry staining in case 2. **a**, **b** HE staining shows a large number of fusiform tumour cells arranged in a palisade-like pattern. **c** The tumour sections were positive for S-100 by immunohistochemistry staining

A 37-year-old man who was diagnosed with LDH was referred to our hospital. He complained of radiating pain in the right lower limb for approximately 9 months. The pain radiated from the posterior aspect of the right thigh to the lateral aspect of the leg and right foot. The persistent and intense pain could be aggravated after hip flexion and slightly relieved after rest or hip overextension. The VAS score for pain was 7/10 at the first visit to the clinic, and the pain could wake the patient up from sleep during the night. Moreover, the pain was combined with numbness of the right lower limb. Physical examination revealed a decreased tingling sensation of the right dorsolateral foot and sole skin, grade 3/5 weakness of the quadriceps femoris, grade 2/5 weakness of the extensor hallucis longus and extensor digitorum longus and a positive straight leg raise test. The muscular tension, knee reflex and Achilles tendon reflex were normal, and he had a negative Babinski sign, Chaddock sign, Gordon sign and Oppenheim sign, and there was no tenderness in the lumbar spine. He had received conservative treatment, which included analgesic drugs, acupuncture treatment and local nerve blocks for approximately 6 months in Chengde Central Hospital. Thereafter, the symptoms were slightly alleviated (VAS = 6/10). The patient also underwent a lumbar MRI examination in Chengde Central Hospital that showed L4-5 intervertebral disc expansion. Although LDH was diagnosed in Chengde Central Hospital, the radiological imaging results were inconsistent with the symptoms and signs. To further explore the cause of the radiating pain in the lower limb radiation, the patient was transferred to our hospital and received a detailed physical examination. Fortunately, a soft tissue mass in the area where the sciatic nerve runs in the middle thigh was palpated. The tumour size was approximately 50 × 30 mm, the boundary was not clear, and there was a positive Tinel’s sign. Subsequently, the patient underwent an enhanced MRI scan that revealed a fusiform tumour in the femoral region. The T1-weighted image showed a nonuniform low-intensity signal, while the T2-weighted image showed a mixed low- and high-intensity signal (Fig. 4). The patient underwent excision biopsy on 23 April 2015. HE staining showed cells with enlarged nuclei and variable degrees of nuclear pleomorphism and mitosis. Immunohistochemical staining showed smooth muscle actin (SMA) (+), neuron-specific enolase (NSE) (+), S-100(+), and vimentin (+). The pathology indicated that the mass was a malignant peripheral nerve sheath tumour (Fig. 5). Then, the patient received cisplatin (DDP) chemohyperthermia on 4 May 2015. After approximately 6 cycles of DDP chemohyperthermia, the tumour completely regressed.

## Discussion and conclusions

Common clinical manifestations of LDH include typical dermatomal pain, increased pain on coughing, leg coldness, sneezing, paresis, straining, absence of knee or ankle tendon reflex, a finger-floor distance of more than 25 cm and a positive straight leg raise test, mostly at the L4-5 or L5-S1 level [3]. On rare occasions, a sciatic nerve tumour can also cause similar clinical manifestations. Filler et al. reported that among patients with sciatica of non-disc origin, the incidence rate of pain caused by sciatic nerve tumours was 1.7% [16, 17]. As previously reported in the literature [4–6, 10, 11], the reason for misdiagnosis as LDH is the lack of mastering of the exact physical examination method and an overdependence on the radiological examination. In addition, tumour-associated sciatica may be prematurely misinterpreted as sciatica caused by LDH because the imaging findings of elderly patients often show clinically asymptomatic degenerative disc changes [18, 19]. As early as 1990, E. Bes, Konakli et al. reported that two femur osteosarcoma patients were misdiagnosed with LDH [6]. The author suggested that all clinicians should be aware of the possibility of sciatic nerve tumours, especially when the imaging findings seem inadequate for explaining the patient’s symptoms and signs [6].

To date, the relevant reports on sciatic nerve tumours misdiagnosed as LDH are case reports [4–10, 14, 20, 21]. We systematically reviewed these reported cases. The subtypes of tumours misdiagnosed as LDH included schwannoma, low-grade sarcoma, osteosarcoma, extraosseous Ewing sarcoma, lipoma, malignant peripheral nerve sheath tumour, and diffuse large B-cell lymphoma. Sciatica and a palpable mass are described as the most common clinical manifestations in the reported cases, followed by atrophy, tenderness on deep intragluteal palpation, positive Tinel’s sign, positive Laseque’s sign, plantar flexion weakness, and decreased ankle reflex. Surgical treatment is the most common treatment, followed by radiotherapy, chemotherapy, and neurolysis (Table 1). Similar to our cases, the two patients presented with sciatica; one of them received surgery and chemotherapy, and the other only received chemotherapy. In the literature review, we also found that in non-discogenic sciatica cases, the patients usually present with such clinical manifestations and that many nerve roots, not just one, can be affected. Thus, we suggest that to accurately detect the cause of nerve root lesions, patients must receive a detailed physical examination and gluteal and/or pelvic MRI.

**Table 1:** Summary of the reported cases of sciatic nerve tumours misdiagnosed as LDH

Author [ref.]	Sex	Age	Clinical presentation	Diagnosis	Misdiagnosis	Treatment	Follow-up	T1WI	T2WI	CE
Wei-Ting Wu [4]	male	27	Sciatica, Tinel’s sign present	Schwannoma	LDH	neurolysis	recure	none	Hyper	Rim
Fernando Guedes [5]	female	37	Sciatica, TDIGP, Tinel’s sign present	low-grade sarcoma	LDH	surgery	stable for 2 years	none	none	none
E.Bes¸konaklı [6]	female	21	Sciatica,a positive Laseque’s sign, plantar flexion weakness, numbness, and decreased achille reflex	osteosarcoma	LDH	surgical and medical treatment	died of pulmonary metastases 8 months later.	none	none	none
E.Bes¸konaklı [6]	female	55	Sciatica, Laseque’s sign, numbness, plantar flexion weakness,and abolished ankle reflex	osteosarcoma	LDH	surgical and medical treatment	none	none	none	none
Jung Soo Bang [10]	female	56	Sciatica, numbness, decreased ankle reflex	extraosseous Ewing Sarcoma	LDH	surgery	stable for 5 months	Iso	hyper	Rim
Peng Wang [11]	female	46	Sciatica	malignant peripheral nerve sheath tumor	LDH	surgery	pulmonary metastases 3 years	none	none	none
A. Peters [13]	none	67	Sciatica	lipoma	LDH	surgery	none	none	none	none

*Note*: *Hypo* hypointense, *Hyper* hyperintense, *Iso* isointensive, *CE* contrast enhancement, *TDIGP* tenderness on deep intragluteal palpation, *LDH* intervertebral disc herniation

To avoid misdiagnosis, it is essential to recognize the clinical manifestations of non-discogenic sciatica causes. First and foremost, for people over 50 years old, if the clinical manifestation is progressive and if severe pain at night is present without remission, the acute sciatica syndrome must be suspected to be caused by a malignancy [21, 22]. In our experience, these patients were more often accompanied by weight loss and a more positive straight leg raise test than those with LDH, and the pain was not related to the position of the lumbar spine. The sciatica caused by sciatic nerve tumours will worsen when hip flexion occurs and is not relieved after rest. Sometimes the pain is only distal, without any radicular distribution [23–25]. Neurological tumours causing motor deficits should always raise the suspicion of a malignant tumour [26, 27]. Sen-Yung Liu et al. [28] suggested that in patients with both sciatic nerve tumours and LDH, when the symptoms gradually worsen after conservative treatment, we must consider the cause of the pain to be sciatic nerve tumours. As case 1 reflected, the patient was misdiagnosed with LDH and received posterior lumbar discectomy and interbody fusion surgery. However, the symptoms were not alleviated. From the above, we can observe that when patients present with sciatica and imaging findings of the lumbar spine that do not justify a discogenic source, the cause should be considered non-discogenic, and the patients should receive gluteal and/or pelvic MRI to exclude tumours. In addition, it is necessary to recognize the importance of basic physical examinations for obtaining a correct diagnosis. Our patient was subjected to inappropriate surgical treatment due to the lack of a gluteal physical examination before the operation. Furthermore, the overestimation of the diagnostic value of radiological examinations for LDH may result in misdiagnosis and wrong treatments. Second, the radiological imaging findings were inconsistent with the patient’s symptoms and signs, and we should explore the pathogenesis to avoid a misdiagnosis. For example, in case 2, we found inconsistencies between the imaging and clinical manifestations, and a malignant peripheral nerve sheath tumour (MPNST) was found. Peter Y. M. Woo et al. [29] reported a rare case of a patient with schwannoma growing on the intrapelvic sciatic notch. The author found that during digital rectal examination, the patient developed a positive Tinel’s sign, presenting with sciatica. Therefore, we advise that an MRI of the gluteus region and physical examination along the sciatic nerve course should be performed in patients with non-discogenic sciatica to exclude more serious causes, and digital rectal examination can be performed if necessary. Finally, the typical symptoms of endometriosis are cyclical sciatica without low back pain. Such cases of sciatica are related to menstruation [30]. Fernando Guedes et al. [5] reported an endometriosis patient who complained of sciatica, which was exacerbated by sitting and relieved by lying down. This patient responded well to hormonal therapy.

In conclusion, we reported two cases of soft tissue masses that were misdiagnosed as LDH. Although diffuse large B-cell lymphoma and schwannoma are very rare in clinical work, they are of great significance in differentiating between discogenic and non-discogenic sciatica. When a patient with sciatica presents with pain while sitting, marked tenderness to deep intragluteal palpation, a positive Tinel’s sign, and imaging findings that seem inadequate for explaining the patient’s symptoms and signs, the patient should receive gluteal and/or pelvic MRI, even when no palpable mass is evident. In patients with both sciatic nerve tumours and LDH, when the symptoms gradually worsen after conservative treatment, we must consider that the cause of the pain is sciatic nerve tumours.

## Data Availability

The datasets used and/or analyzed during the current study are available from the corresponding author on reasonable request. Readers can access the data and material supporting the conclusions of the study by contacting Li Zhao at 654720450@qq.com.

## Abbreviations

LDH: Lumbar disc herniation
MPNST: Malignant peripheral nerve sheath tumour
VAS: Visual analogue scale
MDT: Multidisciplinary team
PET-CT: Positron emission tomography-computed tomography
HE: Haematoxylin-eosin
MUM1: Multiple myeloma oncogene 1
Bcl-6: B-cell lymphoma-6
RCHOP: Cycle rituximab, cyclophosphamide, hydroxy daunomycin, oncovin, prednisone
SMA: Smooth muscle actin
NSE: Neuron-specific enolase
MRI: Magnetic resonance imaging
EMG: Electromyogram
